# Integrated transcriptomic and metabolomic analysis provides insight into the pollen development of CMS-D1 rice

**DOI:** 10.1186/s12870-024-05259-2

**Published:** 2024-06-12

**Authors:** Jie Wang, Suping Ying, Weixiong Long, Lihua Luo, Mingjuan Qian, Wei Chen, Laiyang Luo, Weibiao Xu, Yonghui Li, Yaohui Cai, Xiaojue Peng, Hongwei Xie

**Affiliations:** 1 Jiangxi Super-Rice Research and Development Center, Jiangxi Provincial Key Laboratory of Rice Germplasm Innovation and Breeding, Jiangxi Academy of Agricultural Sciences, National Engineering Research Center for Rice, Nanchang, 330200 China; 2 Key Laboratory of Molecular Biology and Gene Engineering of Jiangxi Province, College of Life Science, Nanchang University, Nanchang, 330031 China

**Keywords:** Cytoplasmic male sterility, CMS-D1, Transcriptome, Metabolome, Jasmonic acid

## Abstract

**Background:**

Cytoplasmic male sterility (CMS) has greatly improved the utilization of heterosis in crops due to the absence of functional male gametophyte. The newly developed sporophytic D1 type CMS (CMS-D1) rice exhibits unique characteristics compared to the well-known sporophytic CMS-WA line, making it a valuable resource for rice breeding.

**Results:**

In this research, a novel CMS-D1 line named Xingye A (XYA) was established, characterized by small, transparent, and shriveled anthers. Histological and terminal deoxynucleotidyl transferase-mediated dUTP nick-end labeling (TUNEL) assays conducted on anthers from XYA and its maintainer line XYB revealed that male sterility in XYA is a result of delayed degradation of tapetal cells and abnormal programmed cell death (PCD) of microspores. Transcriptome analysis of young panicles revealed that differentially expressed genes (DEGs) in XYA, compared to XYB, were significantly enriched in processes related to chromatin structure and nucleosomes during the microspore mother cell (MMC) stage. Conversely, processes associated with sporopollenin biosynthesis, pollen exine formation, chitinase activity, and pollen wall assembly were enriched during the meiosis stage. Metabolome analysis identified 176 specific differentially accumulated metabolites (DAMs) during the meiosis stage, enriched in pathways such as α-linoleic acid metabolism, flavone and flavonol biosynthesis, and linolenic acid metabolism. Integration of transcriptomic and metabolomic data underscored the jasmonic acid (JA) biosynthesis pathway was significant enriched in XYA during the meiosis stage compared to XYB. Furthermore, levels of JA, MeJA, OPC4, OPDA, and JA-Ile were all higher in XYA than in XYB at the meiosis stage.

**Conclusions:**

These findings emphasize the involvement of the JA biosynthetic pathway in pollen development in the CMS-D1 line, providing a foundation for further exploration of the molecular mechanisms involved in CMS-D1 sterility.

**Supplementary Information:**

The online version contains supplementary material available at 10.1186/s12870-024-05259-2.

## Background

Heterosis, also known as hybrid vigor, is a well-known phenomenon in the biological realm where the offspring resulting from the crossbreeding of two parents exhibit enhanced qualities compared to their parents, such as increased yield, stress resistance, and adaptability [1]. The application of heterosis in crop plants stands as a significant advancement in modern agriculture. A considerable portion of crops like rice (*Oryza sativa*), maize (*Zea mays*), rape (*Brassica napus*), rye (*Secale cereale*), sorghum (*Sorghum bicolor*), cotton (*Gossypium*), and sunflower (*Helianthus*) are cultivated from hybrid seeds [2]. The utilization of male sterile lines can streamline the complex process of artificial sterilization, leading to improved efficiency in hybrid seed production and yield. As a result, male sterile lines play a pivotal role in the hybrid breeding system. Male sterility encompasses cytoplasmic male sterility (CMS) and genic male sterility (GMS), with CMS involving interactions between mitochondrial and nuclear genomes, while GMS is solely influenced by nuclear genes [3]. Hybrid seed technology based on CMS adopts a three-line system, necessitating the presence of distinct breeding lines: the CMS line, the maintainer line, and the restorer line. In contrast to CMS, most GMS mutants, with the exception of photoperiod- or thermo-sensitive genic male sterile (P/TGMS) lines, are not suitable for hybrid seed production due to challenges in efficiently maintaining their male-sterility traits [4].

The utilization of CMS in rice hybrid production has been widespread, with over 60 CMS lines identified, such as CMS-WA, CMS-HL, CMS-BT, CMS-LD, CMS-CW, CMS-RT102, CMS-RT98, CMS-TA, CMS-MX, CMS-K, CMS-G, CMS-D1, and CMS-FA [5, 6]. These CMS lines can be classified into three main systems based on inheritance patterns, morphology of abortive pollens, and restoration maintenance relationships: CMS-WA (wild abortive), CMS-BT (Boro II), and CMS-HL (Honglian) [5]. These three systems have been extensively utilized in hybrid rice breeding in China and other Asian countries due to their ability to produce higher yields compared to inbred varieties [7]. The genes responsible for CMS in these systems have been successfully identified and characterized. For instance, in CMS-WA rice, the WA352 protein is specifically produced in the anther tapetum at the microspore mother cell (MMC) stage, where it interacts with COX11 to trigger mitochondrion-driven premature tapetal programmed cell death (PCD), leading to sporophytic male sterility [8]. In CMS-BT rice, the *orf79* gene encodes a cytotoxic peptide, and its protein accumulates preferentially in the microspores to cause gametophytic male sterility [7]. Similarly, in CMS-HL rice, the accumulation of ORFH79 in mitochondria during pollen development results in an increase in reactive oxygen species (ROS) and a decrease in the ATP/ADP ratio in anthers, leading to gametophytic male sterility [9]. CMS-D1, a novel sporophytic CMS line derived from Dongxiang wild rice (*Oryza rufipogon* L.), exhibits unique characteristics such as abnormal anther shape and a no-pollen-grain phenotype. Unlike the well-known sporophytic CMS-WA line, fertility restoration for CMS-D1 is challenging with existing restorer and maintainer lines [10]. The male sterility in CMS-D1 rice is caused by the mitochondrial chimeric gene *orf182*, which might disrupt mitochondrial functions by affecting the mitochondrial respiratory chain complex [10]. Fujian abortive CMS (CMS-FA) rice, developed using cytoplasm from common wild rice (*Oryza rufipogon* L.), exhibits stable sporophytic male sterility controlled by the mitochondrial gene *FA182* [6].

The phytohormone jasmonic acid (JA) plays a critical role in various aspects of rice development, such as spikelet development [11–13], floret opening time (FOT) [14], photomorphogenesis [15], juvenile-to-adult phase transition [16], as well as male fertility [17–20]. Research has shown that elevated JA levels led to early anther dehiscence in the *Ostie1* mutant, a GMS line with inviable pollen and early stamen filament elongation [11]. The interaction between EG2/OsJAZ1, the putative JA receptor OsCOI1b, and the transcription factor OsMYC2 suppresses OsMYC2’s role in activating the E-class gene *OsMADS1* during spikelet development [12]. Loss of *OsCOI1b* function has been shown to have a negative impact on spikelet fertility and grain filling [21]. Another jasmonate receptor, OsCOI2, regulates rice male fertility by controlling anther dehiscence and pollen germination rate [18–20]. These findings highlight the distinct regulatory role of JA in rice spikelet development and male sterility. In the PTGMS rice line PA64S, higher JA levels in young spikelets under high temperature conditions (sterile) compared to low temperature conditions (fertile) indicate JA’s significant role in rice pollen fertility in the PTGMS line [17]. In the CMS-WA line ZS97A, a deficiency in JA inhibited lodicule expansion by retarding the accumulation of osmotic regulation substances and water, leading to scattered FOT [14]. In the CMS-HL line Yuetai A (YtA), higher levels of JA precursors, 12-oxophytodienoic acid (OPDA) and OPC-6:0, were observed during the meiosis state and tetrad stage, respectively, compared to the maintainer line YtB [22]. These findings illustrate the diverse roles of JA in different types of rice CMS systems.

Recently bred CMS lines, CMS-D1 and CMS-FA, both carry the CMS gene *orf182* from different wild rice species. The cloning of the restorer gene *OsRf19* and the analysis of the *orf182*/*OsRf19* mechanism present a promising system for future hybrid rice breeding [6, 10]. In this study, a new CMS-D1 line, Xingye A (XYA), was developed. The programmed cell death (PCD) of tapetal cells and microspores in XYA was characterized using the terminal deoxynucleotidyl transferase-mediated dUTP nick-end labeling (TUNEL) assay. Transcriptomic and metabolomics profiles of young panicles were integrated to identify the metabolic pathways associated with fertility in XYA. Interestingly, the JA biosynthesis pathway was found to be involved in the pollen development of XYA.

## Results

### Phenotypic analysis of CMS-D1 line XYA

**Fig. 1:**
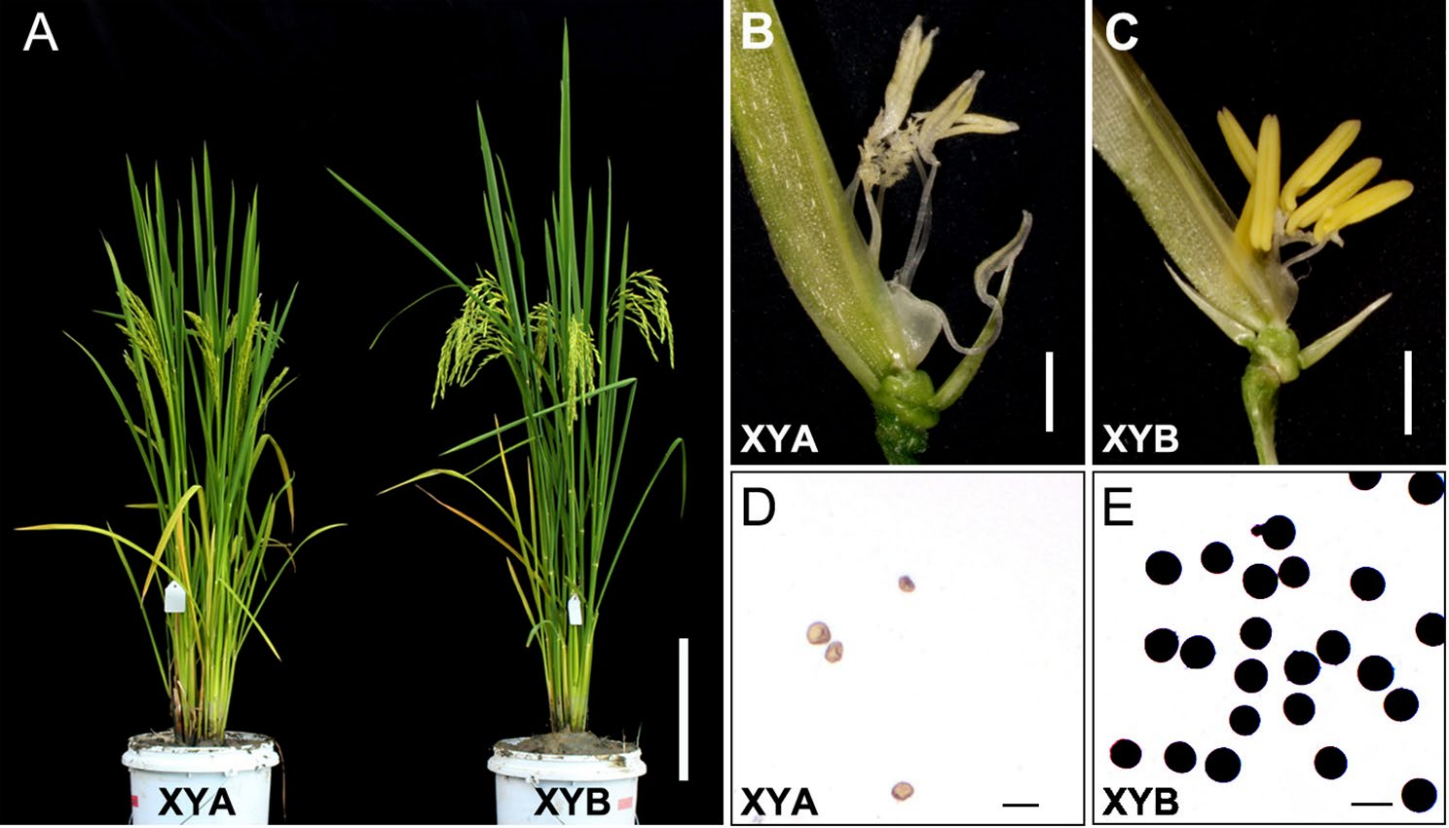
Phenotypic characterization of the CMS-D1 line XYA and its maintainer line XYB. **A**, gross morphologies of XYA and XYB. Scale bar, 20 cm. Anther morphology of XYA (**B**) and XYB (**C**). Scale bars, 1 mm. Pollen morphology of XYA (**D**) and XYB (**E**) matured pollen grains were stained with I_2_-KI. Scale bars, 50 μm

CMS-D1 rice, a sporophytic cytoplasmic male-sterile rice derived from Dongxiang wild rice, contains the mitochondrial chimeric gene *orf182* that is associated with non-pollen type sporophytic male sterility in the CMS-D1 line DPA [10]. However, when DPA was used to produce hybrid rice, low stigma exposure and outcross rates were observed, leading to reduced grain yield. To address these issues, DPA was hybridized with the maintainer line Xingye B (XYB) to create a near isogenic line (NIL) named XYA (Supplemental Figure S1). Compared to XYB, XYA exhibited obvious sterility during the rice grain-filling stage (Fig. 1A). An analysis of anther morphology revealed that XYA had smaller, more transparent, and shrunken anthers compared to XYB (Fig. 1B-C). Additionally, XYA produced very few pollen grains that could not be stained by I_2_-KI, in contrast to the normal fertility of XYB (Fig. 1D-E).

### Histological and TUNEL assays in XYA

**Fig. 2:**
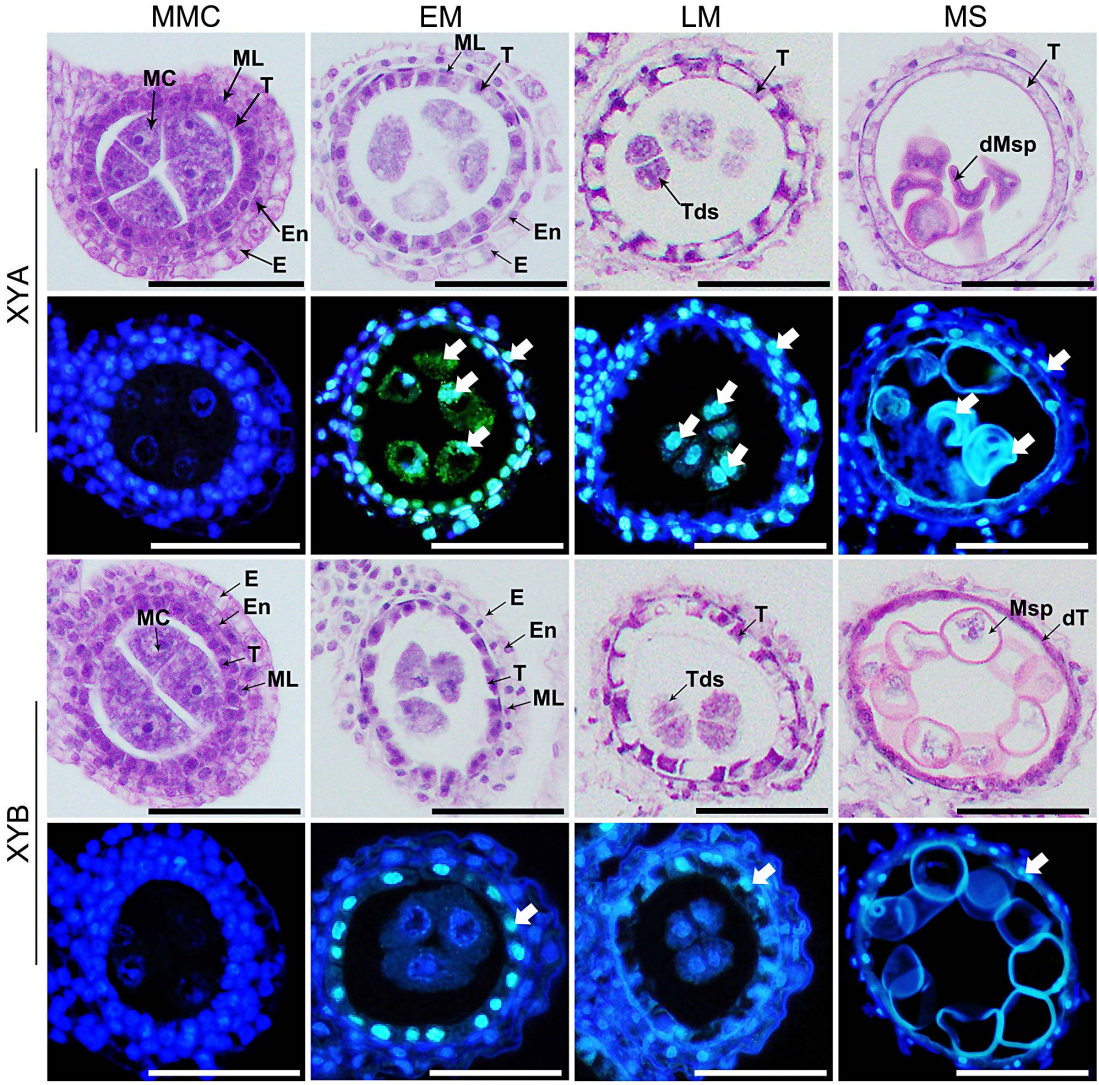
Histological and TUNEL assays of XYA and XYB anthers. The first and third lines, traverse sections of XYA and XYB anthers, respectively. The second and fourth lines, TUNEL assays of XYA and XYB anthers, respectively. The blue signal corresponds to 4’,6-diamidino-2-phenylindole (DAPI) staining, while cyan fluorescence results from the merged signal from TUNEL (green) and DAPI staining (blue). Scale bars, 50 μm. MMC, microspore mother cell stage. EM, early meiotic stage. LM, late meiotic stage. MS, microspore stage. E, epidermis. En, endothecium. ML, middle layer. T, tapetum. MC, microspore mother cell. Tds, tetrads. Msp, microspore. dT, degraded tapetum. dMSP, degraded microspore

The programmed cell death (PCD) process of anther tapetum cells is crucial for anther development, as both premature and delayed PCD can result in male sterility [23]. We examined XYA anther development through anther transverse sections, revealing distinct cellular abnormalities compared to XYB. At the microspore mother cell stage (MMC), no defects were observed in XYA anthers. During the meiosis stage, tapetal cells in XYB anthers underwent thinning, condensation, and gradual degeneration before microspore formation (Fig. 2). In contrast, tapetal cells in XYA anthers did not degrade, leading to a thicker anther wall and reduced pollen grain production (Fig. 2).

A TUNEL assay showed that in XYB anthers, a positive TUNEL signal was detected in tapetal cells during the meiosis stage, gradually weakening before microspore formation (Fig. 2). Conversely, XYA tapetal cells and microspore mother cells (MC) exhibited a strong TUNEL signal during early meiosis (EM) and this persisted in tapetal cells and microspores (Msp) until the microspore (MS) stage (Fig. 2). These findings suggest that delayed PCD in the tapetum layer and abnormal PCD of microspores in XYA anthers during meiosis are the primary causes of male sterility.

### Transcriptome analysis of XYA and XYB

To investigate changes in transcription in CMS-D1 line, RNA-seq analysis was conducted on rice young panicles from both XYA and XYB. A total of 12 libraries were constructed and sequenced using the Illumina HiSeqTM 4000 platform. The high-throughput RNA-seq generated 51.7 to 58.7 million raw reads for each sample. After filtering out low-quality reads from the raw data, the number of clean reads was higher than 50.9 million for each sample (Supplemental Table S1). These clean reads were then mapped to the reference genome with match ratios in the range of 94.50–95.06%. The percentage of Q30 bases in each sample ranged from 92.92 to 93.47%, and the GC content ranged from 49.71 to 50.77% (Supplemental Table S1).

**Fig. 3:**
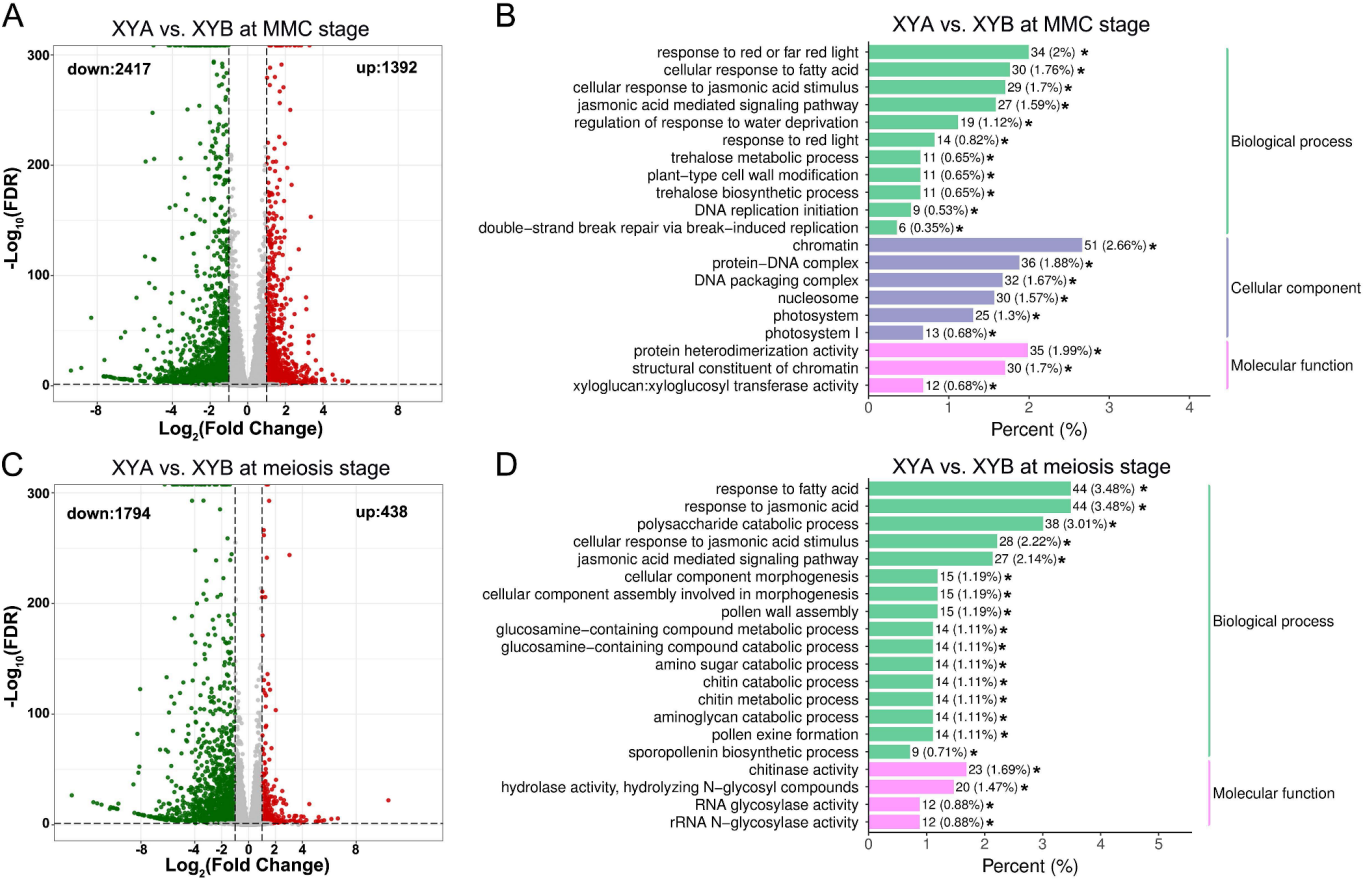
RNA-seq analysis of XYA and XYB. (**A**) Volcano map of DEGs in XYA vs. XYB at MMC stage. (**B**) Significantly enriched GO terms of DEGs in XYA vs. XYB at MMC stage. (**C**) Volcano map of DEGs in XYA vs. XYB at meiosis stage. Red dots and green dots in (**A**) and (**C**) indicate upregulated expression genes and downregulated expression genes, respectively. (**D**) Significantly enriched GO terms of DEGs in XYA vs. XYB at meiosis stage. “*” in (**B**) and (**D**) indicates *P* value < 0.05

Differential expression analysis comparing XYA and XYB at microspore mother cell (MMC) stage revealed a total of 3809 differentially expressed genes (DEGs). Among these, 2417 DEGs exhibited down-expression while 1392 DEGs showed up-expression (Fig. 3A, Supplemental Table S2). Gene Ontology (GO) term analysis demonstrated that the 3809 DEGs were significantly enriched in biological process such as response to red or far red light, cellular response to fatty acid, cellular response to jasmonic acid (JA) stimulus, JA-mediated signaling pathway, and regulation of response to water deprivation (Fig. 3B). Additionally, the cellular component including chromatin, protein-DNA complex, DNA packaging complex, and nucleosome, as well as molecular function such as protein heterodimerization activity and structural constituent of chromatin, were also significantly enriched (Fig. 3B), suggesting potential differences in DNA replication between XYA and XYB during the MMC stage. Kyoto Encyclopedia of Genes and Genomes (KEGG) pathway enrichment analysis revealed significant impacts of the DEGs on plant hormone signal transduction and metabolic pathways at the MMC stage (Supplemental Figure S2A). When comparing XYA to XYB (referred to as XYA vs. XYB) at the meiosis stage, 2232 DEGs were identified, with 438 up-regulated and 1794 down-regulated DEGs (Fig. 3C, Supplemental Table S3). GO term analysis indicated significant enrichment of DEGs in biological processes such as response to fatty acid, response to JA, polysaccharide catabolic process, JA mediated signaling pathway, cellular component morphogenesis, pollen wall assembly, and chitinase activity (Fig. 3D). KEGG enrichment analysis further revealed significant enrichment of DEGs in cutin, suberine, and wax biosynthesis, metabolic pathways, biosynthesis of secondary metabolites, and phenylpropanoid biosynthesis (Supplemental Figure S2B). These results suggest that DEGs in XYA vs. XYB at the meiosis stage primarily respond to fatty acid, JA, and pollen wall assembly.

### Specific DEGs in XYA vs. XYB at the MMC and meiosis stages

**Fig. 4:**
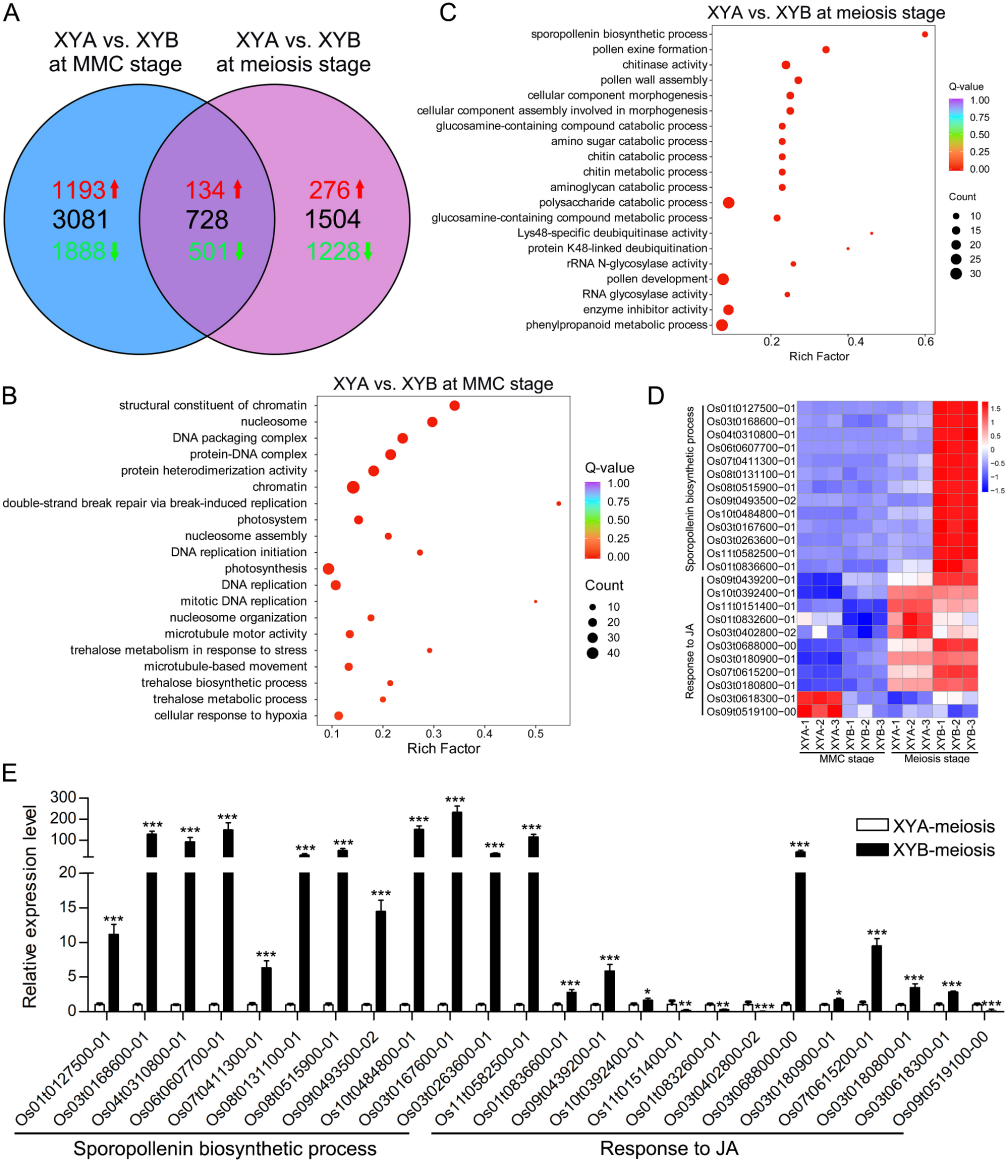
Unique DEGs analysis of XYA and XYB. (**A**) Venn diagram of DEGs. (**B**) GO enrichment analysis of 3081 specific DEGs in XYA vs. XYB at MMC stage. (**C**) GO enrichment analysis of 1504 specific DEGs in XYA vs. XYB at meiosis stage. (**D**) Heatmap illustrating the Log_2_(fold change) of DEGs related to sporopollenin biosynthetic process and JA mediated-signaling pathway. (**E**) Validation of expression levels of DEGs responsive to JA and involved in sporopollenin biosynthesis at the meiosis stage through RT-qPCR. *, **, and *** indicate significant difference at *P* < 0.05, *P* < 0.01, and *P* < 0.001, respectively

To further analyze DEGs specific to the two different stages, we compared the DEGs between XYA and XYB at both the MMC and meiosis stages. In XYA vs. XYB at the MMC stage, a total of 3081 specific DEGs were identified, with 1193 up-regulated and 1888 down-regulated genes (Fig. 4A). These DEGs were significantly enriched in functions related to chromatin structure, nucleosomes, DNA packaging complexes, and chromatin (Fig. 4B). For XYA vs. XYB at the meiosis stage, we found 1504 specific DEGs, including 276 up-regulated and 1228 down-regulated genes (Fig. 4A). GO analysis revealed enrichment in processes such as sporopollenin biosynthetic process, pollen exine formation, chitinase activity, pollen wall assembly, and cellular component morphogenesis at the meiosis stage (Fig. 4C). Additionally, a total of 728 overlapping DEGs were identified between the two stages. Among these, 134 genes were up-regulated and 501 genes were down-regulated at both stages, while 93 genes exhibited opposite expression trends at the two stages (Fig. 4A). These overlapping DEGs were significantly enriched in various biological processes, including cellular response to JA and fatty acid, JA-mediated signaling pathway, and phosphorelay signal transduction system (Supplemental Figure S3). These findings suggest that the specific DEGs in XYA vs. XYB are involved in distinct biological processes at different stages, such as cell division at the MMC stage and pollen development at the meiosis stage.

The heatmap analysis of DEGs associated with sporopollenin biosynthetic process and response to JA revealed that all DEGs related to sporopollenin biosynthesis were down-regulated in XYA at the meiosis stage. However, DEGs involved in JA response displayed varied expression patterns during the meiosis stage (Fig. 4D). Detail information of DEGs linked to sporopollenin biosynthesis and JA response can be found in Supplemental Table S4. The expression levels of DEGs response to JA and involved in sporopollenin biosynthesis during the meiosis stage were confirmed through RT-qPCR. The results demonstrated that the gene expression patterns in XYA compared to XYB during the meiosis stage were in agreement with the FPKM obtained from RNA-seq analysis (Fig. 4E). Based on these results, it can be inferred that the inhibition of sporopollenin synthesis during the meiosis stage may result in defects in pollen wall formation, leading to compromised or reduced pollen development in XYA.

### Metabolic analysis of XYA and XYB

**Fig. 5:**
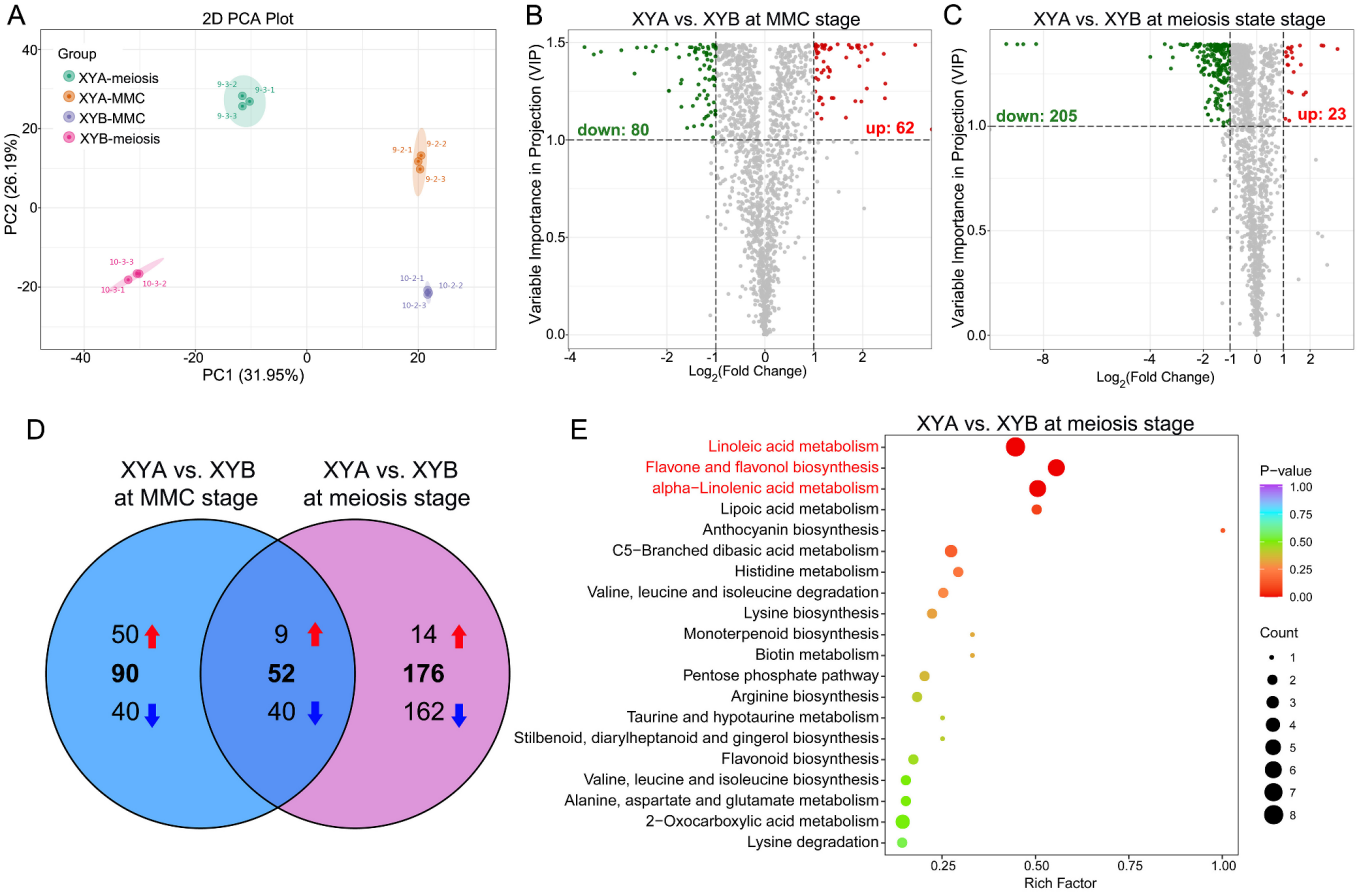
Metabolome analysis of XYA and XYB. (**A**) PCA of the metabolic profiles of XYA and XYB at different stages. Volcano map of DAMs in XYA vs. XYB at MMC stage (**B**) and meiosis state (**C**). Each dot in the volcano map represents a metabolite, where green dots represent down-regulated differential metabolites, red dots represent up-regulated differential metabolites, and gray dots represent metabolites that are detected but not significantly different. (**D**) Venn diagram of DAMs in XYA vs. XYB at different stages. (**E**) KEGG enrichment analysis of 176 specific DAMs at meiosis stage. Pathways highlighted in red represent *P* value < 0.05

The comparison of DEGs in XYA vs. XYB at both MMC and meiosis stages revealed significant enrichment in metabolic pathways according to KEGG analysis (Supplemental Figure S2). Subsequently, metabolic profiles were compared between XYA and XYB, with principal component analysis (PCA) indicating significant differences in metabolites among the XYA-MMC, XYA-meiosis, XYB-MMC, and XYB-meiosis groups (Fig. 5A). A total of 142 differentially accumulated metabolites (DAMs) were identified in XYA vs. XYB at MMC stage, with 62 up-regulated and 80 down-regulated metabolites (Fig. 5B, Supplemental Table S5). Similarly, at the meiosis stage, 228 DAMs were detected in XYA vs. XYB, with 23 up-regulated and 205 down-regulated metabolites (Fig. 5C, Supplemental Table S6). Further KEGG enrichment analysis revealed significant enrichment of DAMs in five pathways at MMC stages, including sphingolipid metabolism, purine metabolism, flavonoid biosynthesis, plant hormone signal transduction, and caffeine metabolism (Supplemental Figure S4A). Similarly, at the meiosis stage, DAMs were significantly enriched in pathways such as flavone and flavonol biosynthesis, α-linolenic acid metabolism, linolenic metabolism, and flavonoid biosynthesis (Supplemental Figure S4B).

Compared to XYB, a total of 176 specific DAMs were identified in XYA at the meiosis stage, with 14 up-regulated and 162 down-regulated. At the MMC stage, XYA had 90 specific DAMs, including 50 up-regulated and 40 down-regulated. Additionally, 52 overlapping DAMs were found in XYA at both MMC and meiosis stages, with 9 up-regulated and 40 down-regulated (Fig. 5D). The 176 specific DAMs at the meiosis stage showed significant enrichment in α-linoleic acid metabolism, flavone and flavonol biosynthesis, and linolenic acid metabolism pathways (Fig. 5E). The 90 specific DAMs at the MMC stage were significantly enriched in plant hormone signal transduction, biosynthesis of amino acids, and sphingolipid metabolism pathways (Supplemental Figure S5A). The 52 overlapping DAMs were significantly enriched in flavonoid biosynthesis and glycosylphosphatidylinositol-anchor biosynthesis pathways (Supplemental Figure S5B). Previous studies have reported that sporopollenin is a dimer formed through the polymerization of phenolics and long-chain aliphatic acids [24]. These findings suggest that α-linoleic acid metabolism and linolenic acid metabolism pathways may impact the synthesis of sporopollenin in XYA anther at the meiosis stage.

### Integrative analysis of the transcriptome and metabolome

**Fig. 6:**
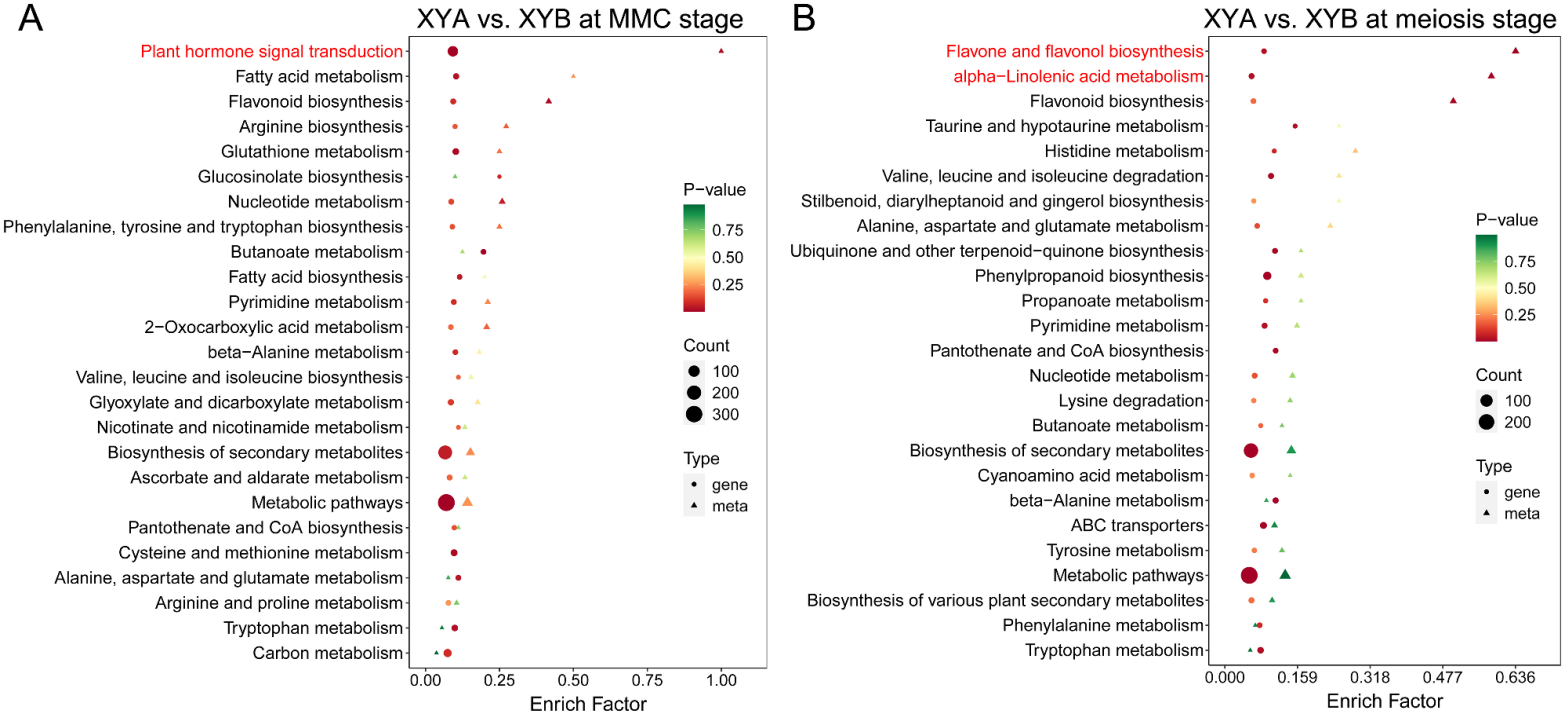
Correlation analysis of DAMs and DEGs in the KEGG pathway. The pathways enriched in XYA vs. XYB at MMC stage (**A**) and meiosis stage (**B**) by transcriptome and metabolome. Only pathways with the top 25 *P*-values in the transcriptome were shown. Pathways highlighted in red indicate *P* value < 0.05 in both transcriptome and metabolome

To elucidate the regulation network involved in pollen development in XYA, an integrated transcriptomic and metabolomics analysis was conducted. At the MMC stage, KEGG analysis revealed significant enrichment of both DEGs and DAMs in the plant hormone signal transduction pathway (Fig. 6A). Specifically, only the JA metabolic pathway showed significant enrichment in both the transcriptome and metabolome (Supplemental Figure S6). During the meiosis stage, KEGG analysis revealed a significant enrichment of flavone and flavonol biosynthesis, as well as α-linolenic acid metabolism (Fig. 6B).

**Fig. 7:**
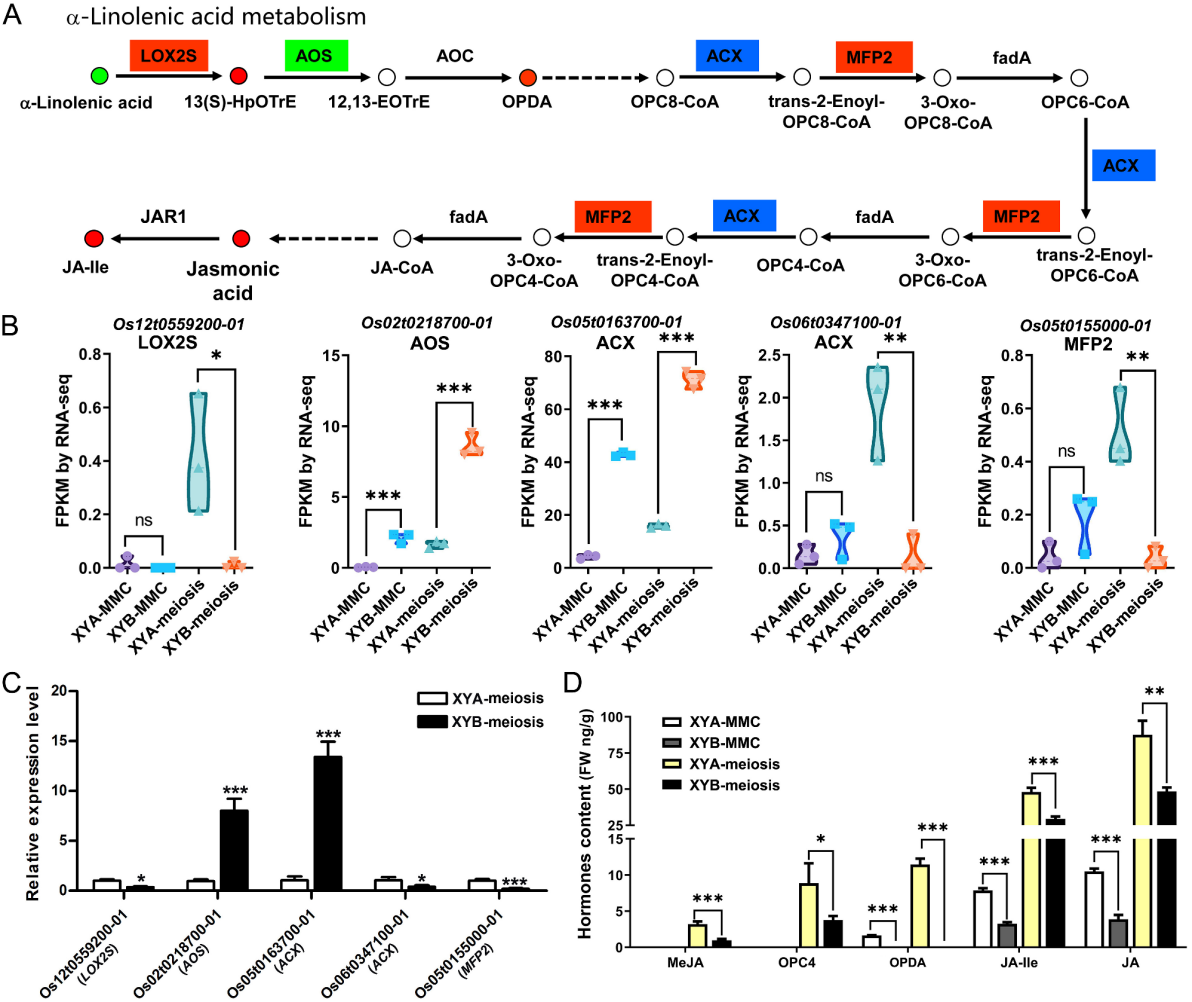
Gene and metabolite changes in α-Linolenic acid metabolism. (**A**) The metabolic pathway of JA synthesis from α-linolenic acid is depicted, with circles representing metabolites and boxes representing genes. Red indicates up-regulated genes/metabolites, green indicates down-regulated genes/metabolites, and blue indicates both up-regulated and down-regulated genes/metabolites. LOX2S, lipoxygenase. AOS, allene oxide synthase. AOC, allene oxide cyclase. ACX, acyl-CoA oxidase. MFP2, multifunctional protein. (**B**) FPKM of DEGs in JA synthesis pathway. (**C**) Expression levels of *LOX2S*, *AOS*, *ACX* and *MFP2* genes at meiosis stage were verified by RT-qPCR. (**D**) Detection of JAs content in XYA and XYB at MMC and meiosis stages. MeJA, Methyl jasmonate. OPC4, 3-oxo-2-(2-(Z)-Pentenyl) cyclopentane-1-butyric acid. OPDA, 12-Oxophytodienoic acid. JA-lle, Jasmonoyl-L-isoleucine. JA, Jasmonic acid. *, **, and *** indicate significant difference at *P* < 0.05, *P* < 0.01, and *P* < 0.001, respectively

The JA biosynthetic pathway was found to be crucial for male sterility [18–20]. Within the α-linolenic acid metabolism, α-linolenic acid undergoes transformation by key enzymes or proteins such as lipoxygenase (LOX2S), allene oxide synthase (AOS), allene oxide cyclase (AOC), acyl-CoA oxidase (ACX), and multifunctional protein (MFP2) to ultimately produce JA (Fig. 7A). During the meiosis stage, despite the lower α-linolenic acid content in XYA compared to XYB, the FPKM values obtained from RNA-seq analysis revealed a significant increase in the expression of *LOX2S* gene, *ACX* gene, and *MFP2* gene (Fig. 7B), leading to higher levels of JA and JA-Ile in XYA through metabolic processes (Fig. 7A). The expression levels of *LOX2S*, *AOS*, *ACX*, and *MFP2* between XYA and XYB at meiosis stage were further confirmed via RT-qPCR, showing consistency with the FPKM values from RNA-seq (Fig. 7C). The JA content was also verified using LC-MS/MS analysis. The results demonstrated higher levels of OPDA, JA-Ile, and JA in XYA compared to XYB at the MMC stage, and elevated levels of MeJA, OPC4, OPDA, JA-Ile, and JA in XYA compared to XYB at the meiosis stage (Fig. 7D). Based on these findings, it is proposed that the increased JA levels in the CMS-D1 line may be crucial for pollen development in XYA.

## Discussions

In seeds plants, after anther morphogenesis is complete, the meiotic cells at the center of each anther lobe are surrounded by four somatic layers: the epidermis, endothecium, middle layer, and tapetum [25, 26]. The tapetum, the innermost sporophytic layers in the anther wall, directly contacts with the developing gametophytes, providing nutrients to microspores and regulating their release during microgametogenesis. Tapetum cells undergo programmed cell death (PCD) during late pollen development, and any disruptions in this process can affect nutrient supply to microspores, resulting in pollen abortion [25]. In CMS-WA rice ZS97A, tapetum degeneration occurs early in microspore development, while in the maintainer line ZS97B, it starts later, indicating a link between premature tapetal PCD and CMS-WA [8]. In CMS-HL rice YtA, the PCD of microspores, not tapetal cells, leads to gametophytic male sterility [27]. This study shows that delayed degradation of tapetal cells and microspores’ PCD during meiosis are the main factors causing male sterility in CMS-D1 rice XYA. Variations in tapetum and microspore degradation among CMS-D1, CMS-WA, and CMS-HL suggest genetically distinct mechanisms for microspore abortion. These findings highlight the unique characteristics of CMS-D1 rice compared to well-known sporophytic CMS-WA rice.

Pollen grains, the male gametophytes in flowering plants, undergo a delicate and complex development process within the anther. The pollen wall is crucial for protecting the male gametophyte and facilitating fertilization, with its different layers synthesized through distinct metabolic pathways. Lipid components for the pollen wall primarily originate from the sporophytic tapetum [28]. Structurally, the pollen wall consists of intine and exine, with polysaccharides and lipidic sporopollenin being the major components, respectively [26]. Research has indicated that abnormal or delayed PCD of the tapetum can disrupt sporopollenin deposition and the formation of a normal pollen wall [29]. The gene *OsTKPR1* in rice has been found to play a significant role in tapetum PCD and pollen wall formation, with loss of function resulting in male sterility due to delayed tapetum degradation and impaired pollen wall formation [30]. Sporopollenin biosynthesis is closely linked to fatty acid metabolism, and inhibition of sporopollenin synthesis can lead to pollen wall defects [31]. This study identified 2232 DEGs through RNA-seq analysis in XYA vs. XYB at the meiosis stage, with enrichment in processes related to fatty acid response, cellular component morphogenesis, and pollen wall assembly. Additionally, the expression levels of the sporopollenin biosynthetic genes in XYA were significantly lower than those in XYB at the meiosis stage, suggesting that reduced expression of these genes at this stage might result in pollen wall defects, leading to the production of scarce and abortive pollen in the CMS-D1 line XYA. The impact of the CMS-D1 gene *orf182* in mitochondria on the expression of sporopollenin biosynthetic genes in the nucleus is a compelling area for future investigation.

Previous research has demonstrated the crucial role of JA in regulating flower development, particularly in anther development [19, 20, 32]. JA deficiency has been linked to scattered floret opening time in CMS-WA rice Zhenshan 97 A [14]. JA also influences the pollen fertility of PGMS line D52S and PTGMS line PA64S [17]. Similar to the CMS-D1 line XYA, the *Ostie1* mutants also exhibit male sterility with a high accumulation of JA in anthers [11]. However, *Ostie1* mutants show inviable pollen, early stamen filament elongation, and precocious anther dehiscence, which differ from the phenotype of XYA. These findings suggest that JA plays a significant role in rice male sterility and control various traits. The biosynthesis of JA is initiated in plastids by the lipase defective in anther dehiscence 1 (DAD1), which produces α-linolenic acid. Subsequently, some key enzymes involved in JA biosynthesis: 13-lipoxygenases (LOXs), allene oxide synthase (AOS), allene oxide cyclase (AOC), and 12-oxo-phytodienoic acid reductase (OPR) [33]. In rice, JA-deficient mutants also exhibit low fertilization rates and abnormal flower formation, indicating that JA plays a crucial role in flower development and fertility in rice [13, 19, 20, 32]. Our study conducted transcriptomic and metabolomic analyses, which revealed a significant enrichment of the α-linolenic acid metabolism pathway during the meiosis stage. This metabolic pathway ultimately leads to the production of JA. Previous studies have identified *OsAOS2* [34], *OsOPR7* [35, 36], and *OsJAR1* [15] as crucial genes in the JA synthesis pathway. However, our transcriptomic analysis indicated no significant difference in the expression levels of these genes between XYA and XYB at the meiosis stage. Conversely, genes like *LOX2S*, *ACX*, and *MFP2* exhibited higher expression levels in XYA compared to XYB according to RNA-seq analysis, suggesting that *LOX2S*, *ACX*, and *MFP2* play a pivotal role in enhancing JA synthesis by increasing the production of intermediates in the CMS-D1 line. The higher JA level in the CMS-D1 line compared to its maintainer line is different from that in the CMS-WA rice, further distinguishing CMS-D1 rice from sporophytic CMS-WA rice. These findings imply that the JA biosynthesis pathway may be crucial in pollen development of CMS-D1 rice, providing a basis for exploring the connection between the sterility gene *orf182* in CMS-D1 rice and JA synthesis.

## Conclusions

The CMS line is a crucial component in hybrid breeding systems. The novel sporophytic CMS-D1 line, distinct from the well-known sporophytic CMS-WA rice, presents significant breeding potential. This research delved into the distinctive features of sporophytic CMS-D1 rice, with a specific focus on the novel CMS-D1 line XYA and its maintainer line XYB. Histological and TUNEL assays revealed delayed degradation in the tapetum layer and PCD of microspores in XYA anthers during meiosis as the causes of male sterility in CMS-D1 rice. Transcriptome and metabolome analyses revealed complex regulatory changes, with the JA biosynthesis pathway potentially playing a key role in pollen development in XYA. Key pathway genes like *LOX2S*, *ACX*, and *MFP2*, which enhance JA synthesis, were identified as potential targets for regulating pollen development in XYA. These findings provide valuable insights into the pollen development of CMS-D1 rice and establish a foundation for further elucidating the molecular mechanisms behind CMS-D1 sterility.

## Materials and methods

### Plant materials and fertility investigation

The CMS-D1 line XYA was obtained from successive backcrosses between DPA and XYB. In this study, XYA and its maintainer line XYB were chosen for further research. The rice materials were cultivated in the experimental fields of Jiangxi Academy of Agricultural Sciences under natural growing conditions. Anthers of mature florets were collected for pollen fertility using the 1% (w/v) I_2_-KI staining method.

### Histological and TUNEL assays

For microscopic analysis, young panicles and florets in various stages were vacuum infiltrated for 30 min, then fixed with 50% FAA (formaldehyde: glacialacetic acid: 50% ethanol in a 1:1:18 ratio, v/v/v) at 4 °C for 24 h. Subsequently, the samples were embedded in paraffin and cut into 6-µm slices using a rotary microtome. Following hematoxylin-eosin staining, the sections were examined under a standard optical microscope CX-21 (Olympus, Tokyo, Japan).

Paraffin sections of panicles and florets were used for TUNEL assays with an in situ cell death detection kit (Roche, Basel, Swiss Confederation), and analyzed under a fluorescence microscope IX51 (Olympus, Tokyo, Japan).

### Transcriptome analysis

The 1.5 cm young panicles (MMC) and 1.5–4.0 cm panicles (Meiosis) were collected for RNA-seq, metabolome analysis, RT-qPCR experiment, and JA contents detection. Total RNA was isolated using the RNAprep Pure Plant Kit (Tiangen, Beijing, China), and its concentration was quantified with the QubitR RNA Assay Kit in QubitR2.0 Flurometer (Life Technologies, CA, USA). Subsequently, 1 µg of RNA per sample was used for library preparation and sequencing on the Illumina platform by Metware Biotechnology Co. Ltd (Wuhan, China).

The clean reads were aligned to the NIP reference genome using Hisat2 v2.2.0 [7], and gene expression levels were estimated using Fragments Per Kilobase of transcript per Million fragments mapped (FPKM). Differential expression genes (DEGs) were identified using the DESeq2 package with criteria of Benjamini-Hochberg-adjusted *p*-value < 0.05 and |log_2_FoldChange| ≥ 1 were set as criteria. GO enrichment and KEGG enrichment analyses were carried out following the methods described by Wang et al. [37].

### Gene expression analysis by RT-qPCR

The total RNA was prepared following the same procedure as described above for transcriptome analysis. For RT-qPCR validation, cDNA was synthesized using M-MLV reverse transcriptase (Promega). Sequence-specific primers were designed using Primer Premier 5.0 software (Palo Alto, CA, USA). RT-qPCR was performed with the QuantStudioTM Real-Time PCR System (Applied Biosystems, USA) using 2×TB Green Mixture (Takara, Beijing, China). The rice *ACTIN* gene (*LOC_Os03g50885*) was used as the internal reference. Three biological replicates were conducted for each sample, and the relative gene expression levels were determined using the 2^–ΔΔCt^ method [38]. The sequence-specific primers for RT-qPCR were listed in Supplementary Table S7.

### Metabolome analysis

Young panicle tissues were prepared according to the methods outlined in the transcriptome analysis. A total of 12 samples, with three biological replicates per group, were collected for a comprehensive analysis of metabolites using the UPLC-MS/MS platform by Metware Biotechnology Co. Ltd (Wuhan, China). The samples were freeze-dried in a lyophilizer under vacuum, then grinded to powder at 30 Hz for 1.5 min. Each sample, consisting of 50 mg of powder, was then suspended in 1.2 mL of 70% aqueous methanol (v/v) pre-cooled to -20 °C for metabolite extraction. The sample extracts were filtered through a microporous membrane with a pore size of 0.22 μm and stored in injection vials for UPLC-MS/MS analysis. Unsupervised PCA was conducted by statistics function prcomp within R (www.r-project.org). The data was unit variance scaled before unsupervised PCA. Differentially accumulated metabolites (DAGs) for two-group comparison were identified based on VIP > 1 and |Log_2_FC| ≥ 1. For KEGG enrichment analysis, the DAGs were annotated using the KEGG Compound database (http://www.kegg.jp/kegg/compound/), and then mapped to the KEGG Pathway database (http://www.kegg.jp/kegg/pathway.html). The pathways were subsequently analyzed using MSEA (metabolite sets enrichment analysis), with significance being determined by hypergeometric test’s *p*-values.

### Determination of JA contents

To detect JA contents, 1.5 cm young panicles (at MMC stage) and 1.5–4.0 cm panicles (at meiosis stage) of XYA and XYB plants were quickly frozen in liquid nitrogen, ground into powder (30 Hz, 1 min), and analyzed by MetWare (http://www.metware.cn/) on the AB Sciex QTRAP 6500 LC-MS/MS platform. Each 50 mg sample was placed in a 2 mL plastic microtube, frozen in liquid nitrogen, dissolved in 1 mL methanol/water/formic acid (15:4:1, V/V/V). A 10 µL internal standard mixed solution (100 ng/mL) was added for quantification. After vortexing for 10 min and centrifugation at 4 °C for 5 min (12,000 r/min), the supernatant was transferred to clean plastic microtubes, evaporated to dryness, dissolved in 100 µL 80% methanol (V/V), and filtered through a 0.22 μm membrane filter for subsequent LC-MS/MS analysis.

### Statistical analysis

Statistical analysis was conducted using GraphPad Prism 5 (GraphPad Software Inc., La Jolla, CA, USA). Student’s *t*-test was used for the evaluation of the *P*-values. At least three biological replicates were analyzed per condition. Data were presented as means ± SD.

### Electronic supplementary material

Below is the link to the electronic supplementary material.

Supplementary Material 1

Supplementary Material 2

Supplementary Material 3

Supplementary Material 4

Supplementary Material 5

Supplementary Material 6

Supplementary Material 7

Supplementary Material 8

## Data Availability

The original contributions presented in the study are included in the article and Supplementary materials, further inquiries can be directed to the corresponding author. The raw data of RNA-seq generated are deposited to the NCBI SRA database under Bioproject No. PRJNA1091800 (https://www.ncbi.nlm.nih.gov/bioproject/PRJNA1091800).
