# Supplementary material for: Integrated transcriptomic and metabolomic analysis provides insight into the pollen development of CMS-D1 rice
Source: BMC Plant Biol. 2024 Jun 12;24:535. doi: 10.1186/s12870-024-05259-2 (PMC11167768; doi:10.1186/s12870-024-05259-2)
Supplement: Supplementary file 1 — Supplementary Material 1 [file 12870_2024_5259_MOESM1_ESM.docx]

**DPA**

**XYB**

**F_1_**

**XYB**

**×**

**BC_1_F_1_**

**XYB**

**BC_2_F_1_**

**XYB**

**…**

**BC_7_F_1_**

**(XYA)**

**×**

**×**

**×**

**Figure S1.** Construction of the near isogenic line (NIL) for XYA. XYA contains the cytoplasm of DPA in the genetic background of XYB (a maintainer line derived from hybridization of Yuehui520 and Wusansimiao).


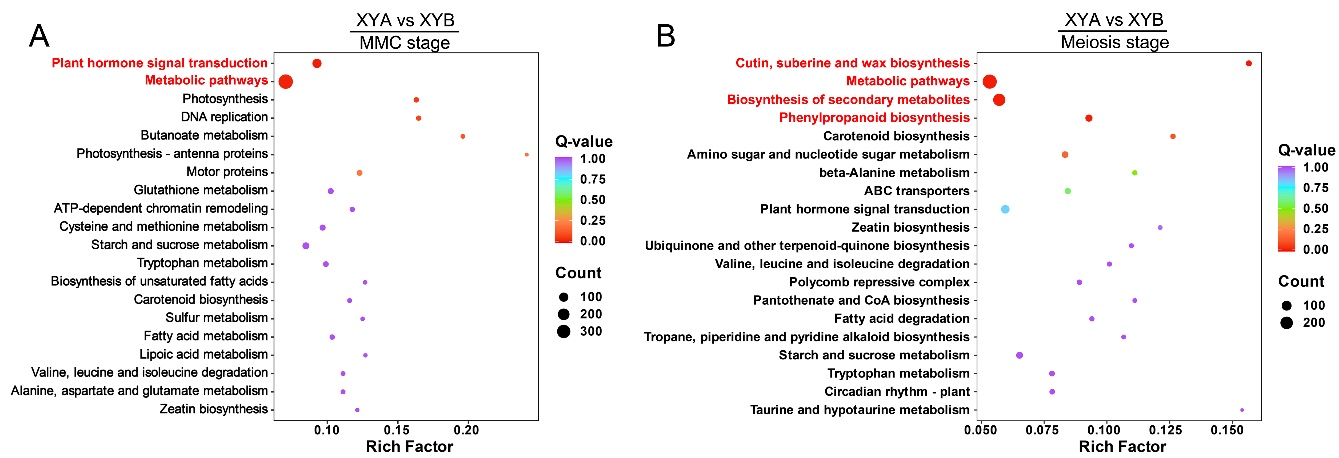


**Figure S2.** KEGG analysis of DEGs in XYA vs. XYB. KEGG enrichment analysis of DEGs in XYA vs. XYB at MMC stage (**A**) and meiosis stage (**B**). Pathways highlighted in red indicate *P* value < 0.05.


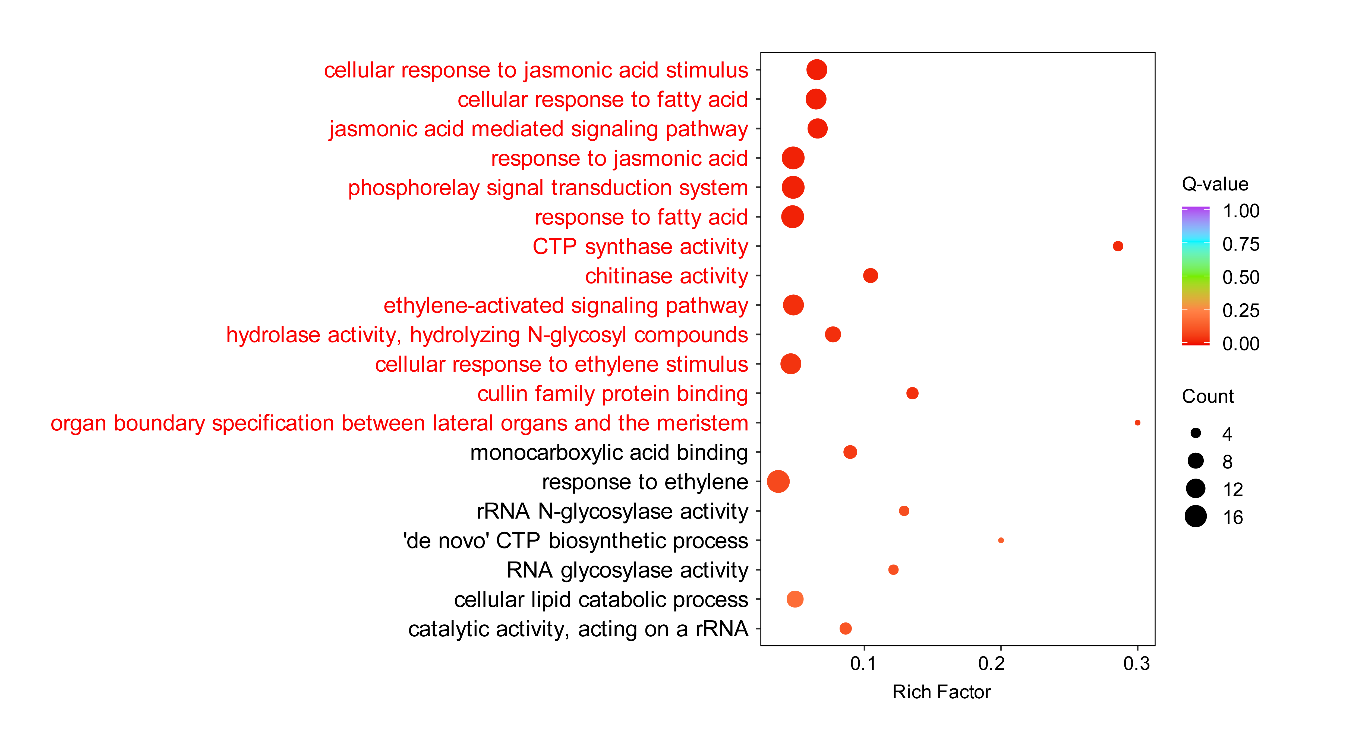


**Figure S3**. GO analysis of the overlapping DEGs in XYA vs. XYB at both MMC and meiosis stages.


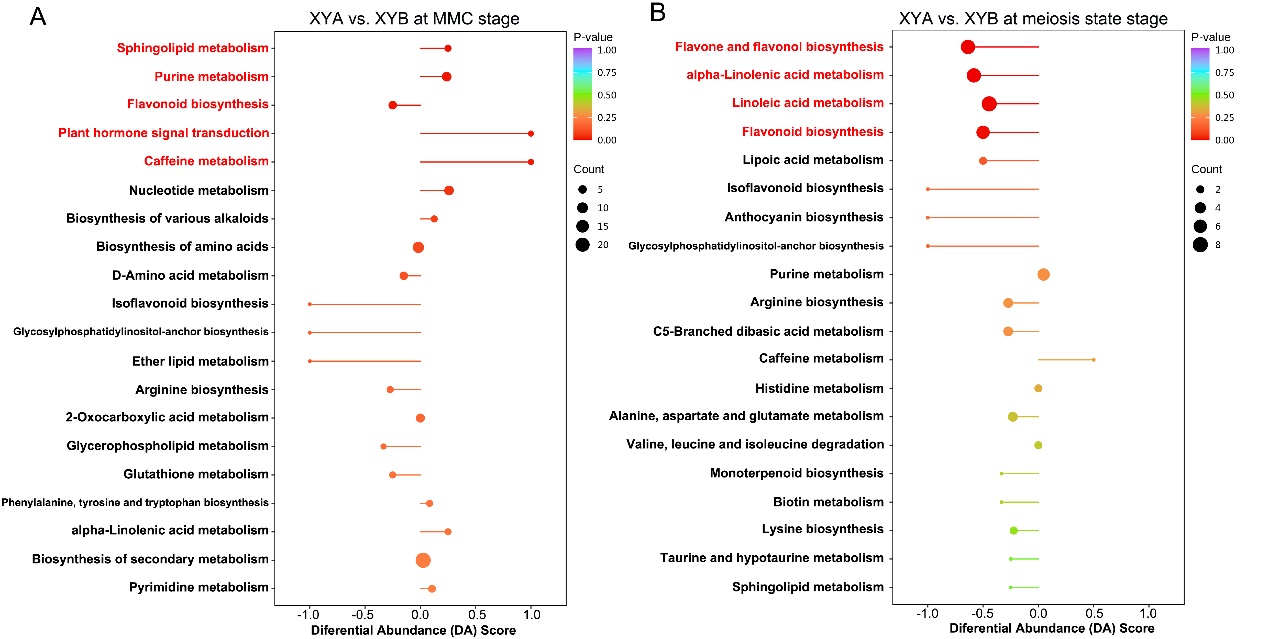


**Figure S4.** Metabolic pathway enrichment analysis of DAMs. KEGG pathway analysis in XYA vs. XYB at MMC stage (A) and meiosis state (B). Dots distributed on the left side of the axis with longer line segments indicates a tendency towards downregulation, while dots on the right side with longer line segments indicate a tendency towards upregulation. Metabolic pathways highlighted in red represent a significant *P* value < 0.05.


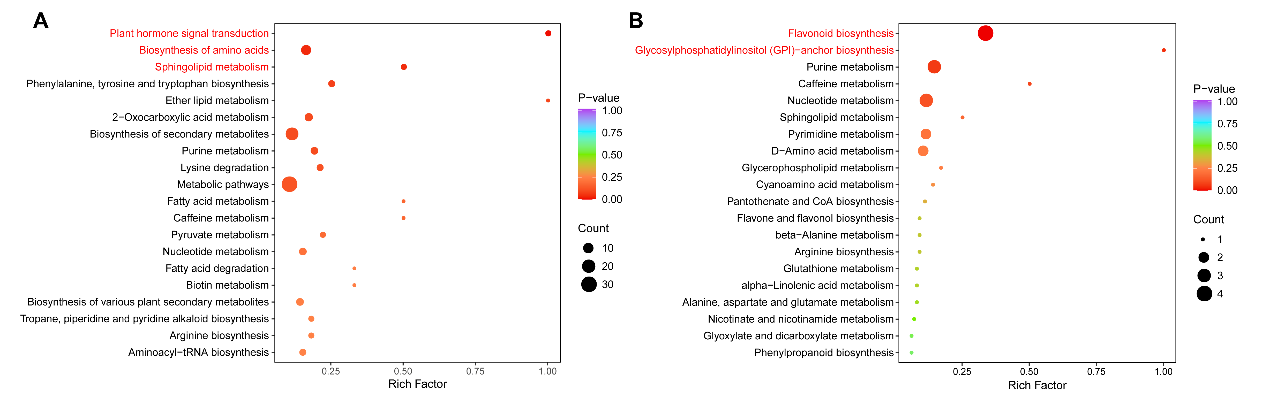


**Figure S5.** Specific DAMs analysis in XYA vs. XYB. **(A)** KEGG enrichment analysis of 90 specific DAMs in XYA vs. XYB at MMC stage. (**B**) KEGG enrichment analysis of 52 overlapping DAMs in XYA vs. XYB. Pathways highlighted in red indicate *P* value < 0.05.


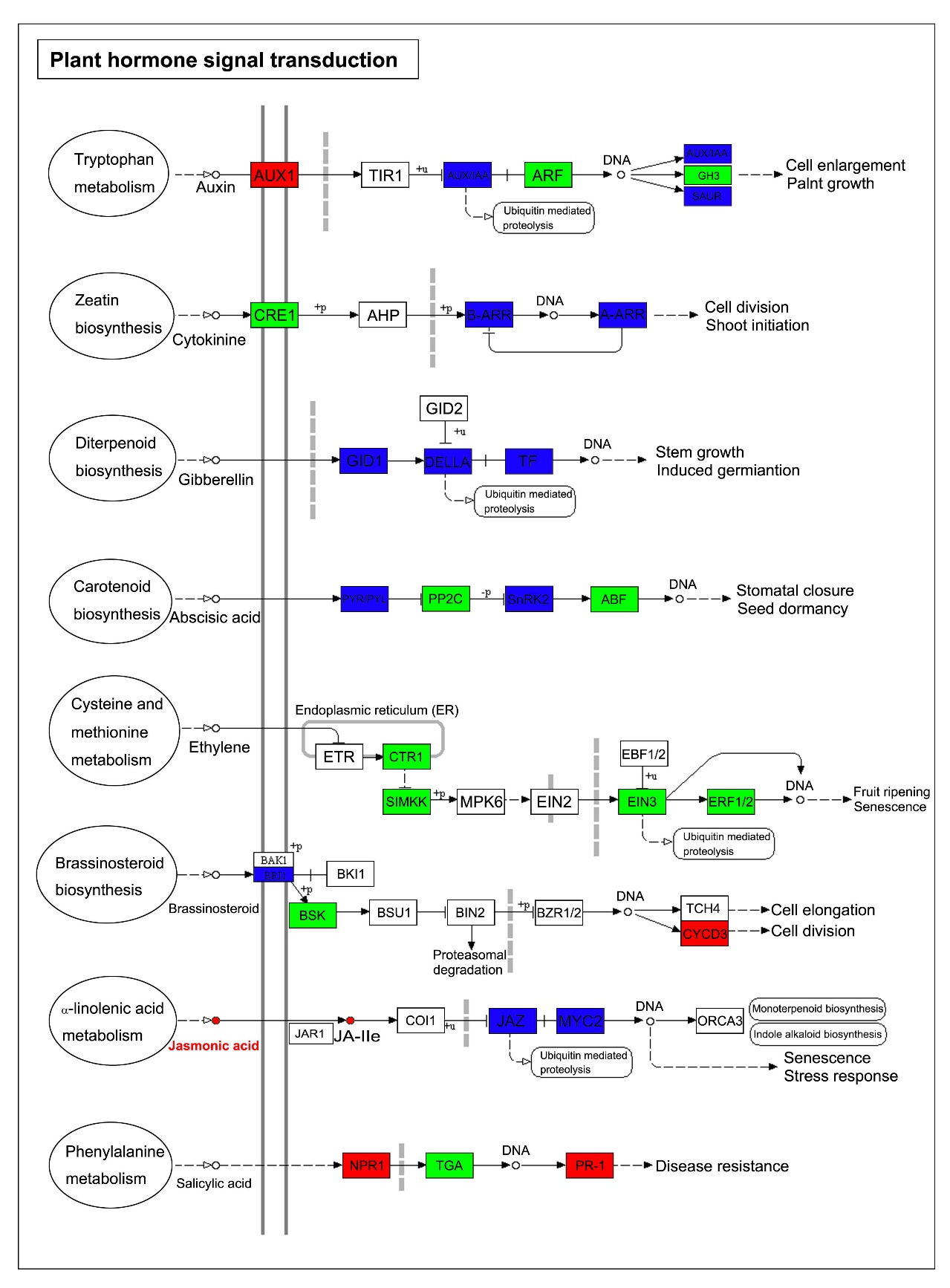


**Figure S6.** Enrichment of both DEGs and DAMs in the plant hormone signal transduction pathway by KEGG analysis.
